# Cognitive Remediation in Patients With Bipolar Disorder: A Randomized Trial by Sequential tDCS and Navigated rTMS Targeting the Primary Visual Cortex

**DOI:** 10.1111/cns.70179

**Published:** 2024-12-20

**Authors:** Hetong Zhou, Minmin Wang, Ting Xu, Xiaomei Zhang, Xudong Zhao, Lili Tang, Pengfei Zhao, Dandan Wang, Jianbo Lai, Fei Wang, Shaomin Zhang, Shaohua Hu

**Affiliations:** ^1^ Department of Psychiatry, The First Affiliated Hospital Zhejiang University School of Medicine Hangzhou China; ^2^ Nanhu Brain‐Computer Interface Institute Hangzhou China; ^3^ Zhejiang Key Laboratory of Precision Psychiatry Hangzhou China; ^4^ Key Laboratory of Biomedical Engineering of Education Ministry, Zhejiang Provincial Key Laboratory of Cardio‐Cerebral Vascular Detection Technology and Medicinal Effectiveness Appraisal, School of Biomedical Engineering and Instrument Science, Qiushi Academy for Advanced Studies Zhejiang University Hangzhou China; ^5^ Westlake Institute for Optoelectronics Westlake University Hangzhou China; ^6^ Department of Psychiatry Huzhou Third Municipal Hospital Huzhou China; ^7^ Early Intervention Unit, Department of Psychiatry The Affiliated Brain Hospital of Nanjing Medical University Nanjing China; ^8^ Functional Brain Imaging Institute Nanjing Medical University Nanjing China; ^9^ Department of Mental Health, School of Public Health Nanjing Medical University Nanjing China; ^10^ Brain Research Institute of Zhejiang University Hangzhou China; ^11^ Zhejiang Engineering Center for Mathematical Mental Health Hangzhou China; ^12^ The State Key Lab of Brain‐Machine Intelligence Zhejiang University Hangzhou China; ^13^ Department of Psychology and Behavioral Sciences Zhejiang University Hangzhou China

**Keywords:** bipolar disorder, cognitive function, primary visual cortex, rTMS, tDCS

## Abstract

**Background:**

Non‐invasive brain stimulation (NIBS), such as transcranial direct current stimulation (tDCS) and repetitive transcranial magnetic stimulation (rTMS), has emerged as a promising alternative in the precise treatment of clinical symptoms, such as the cognitive impairment of bipolar disorder (BD). Optimizing the neurocognitive effects by combining tDCS and rTMS to strengthen the clinical outcome is a challenging research issue.

**Objective:**

In this randomized, controlled trial, we first combined tDCS and neuronavigated rTMS targeting the V1 region to explore the efficacy on neurocognitive function in BD patients with depressive episodes.

**Methods:**

Eligible individuals (*n* = 105) were assigned into three groups, Group A (active tDCS‐active rTMS), Group B (sham tDCS‐active rTMS), and Group C (active tDCS‐sham rTMS). All participants received 3‐week treatment in which every participant received 15 sessions of stimulation through the study, 5 sessions every week, with tDCS treatment followed by neuronavigated rTMS every session. We evaluated the cognitive, emotional, and safety outcomes at week‐0 (w0, baseline), week‐3 (w3, immediately post‐treatment), and week‐8 (w8, follow‐up period). The THINC‐integrated tool (THINC‐it), 17‐item Hamilton Depression Rating Scale, and Young Mania Rating Scale were applied for evaluating the cognitive function and emotional state, respectively. Data were analyzed by repeated measure ANOVA and paired *t*‐test.

**Results:**

Eventually, 32 patients in Group A, 27 in Group B, and 23 in Group C completed the entire treatment. Compared to Groups B and C, Group A showed greater improvement in Symbol Check items (Time and Accuracy) at W3 and Symbol Check Accuracy at W8 (*p* < 0.01). The W0‐W3 analysis indicated a significant improvement in depressive symptoms in both Group A and Group B (*p* < 0.01). Additionally, neuroimaging data revealed increased activity in the calcarine sulcus in Group A, suggesting potential neuroplastic changes in the visual cortex following the electromagnetic stimulation.

**Conclusions:**

These findings provide preliminary evidence that the combination of navigated rTMS with tDCS targeting V1 region may serve as a potential treatment strategy for improving cognitive impairment and depressive symptoms in BD patients.

**Trial Registration:**

Clinical Trial Registry number: NCT05596461

## Graphical abstract

All patients underwent a 3‐week intervention of combined electromagnetic stimulation. Scale assessments and magnetic resonance imaging data collection were performed before and after the intervention, followed by scale assessments at the eighth week.

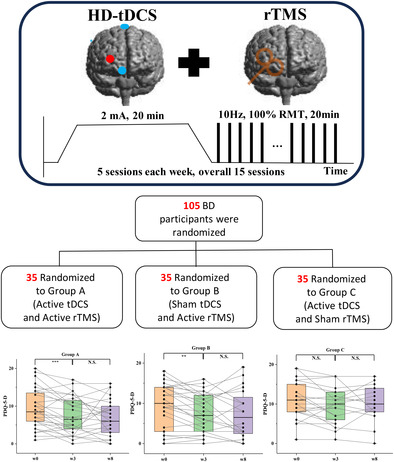

## 1 Introduction

Bipolar disorder (BD) is a chronic, severe psychiatric disease with a profound impact on public health worldwide, boasting a yearly prevalence of 2% [1]. Global disease burden statistics underscored BD as the 17th leading cause of disability globally [2, 3]. Typically occurring in late adolescence or early adulthood, BD is associated with a starkly elevated risk of suicide, estimated to be 20–30 times higher during depressive episodes than the general population [4]. The quintessential clinical hallmark of BD lies in its oscillating emotional states and energy levels, coupled with cognitive and social functioning impairments [5]. Mounting evidence indicate a close relationship between BD and pervasive cognitive deficits, encompassing executive functions, memory, social cognition, and reaction time [6]. The presence of these cognitive deficits notably compromises the life quality of individuals with BD, necessitating the development of effective interventions to ameliorate their clinical symptoms.

The research field of non‐invasive brain stimulation (NIBS), notably including techniques such as transcranial direct current stimulation (tDCS) and repetitive transcranial magnetic stimulation (rTMS), has emerged as a promising frontier in BD treatment [7, 8]. Randomized controlled trials (RCTs) have provided compelling evidence demonstrating the superior efficacy of rTMS compared to placebo in the treatment of BD [9, 10]. Likewise, tDCS displays the potential to enhance cognitive function among bipolar patients [11, 12]. Furthermore, concurrent tDCS and rTMS have been found to enhance modulation effects for cortical activity [13, 14], thereby enhancing working memory [15]. Currently, there are only limited studies suggesting that combined electromagnetic stimulation may enhance the effectiveness of clinical interventions [16, 17, 18]. However, the conjoined impact of tDCS and rTMS on BD patients remains a new area necessitating further exploration.

In addition, the thoughtful choice of stimulation targets significantly influences the treatment outcome of NIBS [19, 20]. Clinical investigations primarily focus on abnormal brain regions or circuits. Notably, cognitive impairment in BD is associated with functional abnormalities within these neural networks [21, 22, 23]. For instance, relative to healthy individuals, BD patients exhibit escalated activation in the ventromedial prefrontal cortex and subgenual anterior cingulate cortex (ACC) during working memory tasks. Moreover, disruptions within critical brain regions implicated in cognitive and emotional processing [24], primarily located within the frontal‐limbic network, are discernible in BD patients [25]. Task‐related functional disparities further materialize in the left superior and right inferior parietal lobules, alongside undue activation in the left medial orbitofrontal cortex. The collective evidence accentuates the potential utility of the dorsolateral prefrontal cortex (DLPFC) as a therapeutic target to enhance cognitive functioning in BD patients [26]. A multitude of clinical investigations correspondingly emphasize interventions within this brain region and its attendant circuits.

Significantly, recent research has unveiled functional connectivity (FC) between the DLPFC and ACC, positing the primary visual cortex (V1) as a prospective target for augmenting neurocognitive function in BD via rTMS [27]. The V1 cortex occupies a pivotal role in visual information transmission [28]. Studies have established a connection between the attenuation of V1 activity following brief visual stimuli and iconic memory. Beyond its role in processing incoming visual stimuli, V1 is also critically involved in visual working memory and attention‐related processes [29, 30]. Alterations within the structure and function of the visual cortex have been implicated in depressive symptoms, with antidepressive agents modulating electrophysiological attributes and neurotransmitter functionality within the visual cortex [31]. Collectively, these findings underscore the crucial role of V1 in modulating emotional symptoms and cognitive functions, highlighting its potential as a novel target for addressing cognitive impairments associated with mental disorders [32]. Our previous study further supported the potential of targeting V1 with rTMS to enhance cognitive function in patients with BD [27]. The current study would handle the modulation mode based on a long‐distance function connectivity and explore establishing the novel strengthening mode combining multiple NIBS technologies.

This integrated approach holds the potential to amplify information importing and interaction between V1 and DLPFC by stimulating the V1 cortex, thus potentially enhancing neurocognitive function in individuals grappling with BD. Consequently, capitalizing on novel stimulus paradigms and targeting strategies, our study aims to leverage NIBS to modulate the V1‐DLPFC function connectivity by sequential tDCS and navigated rTMS, interrogating its impact on neurocognitive function and depressive symptoms among BD individuals.

## 2 Methods and Materials

### 2.1 Trial Design

The trial was conducted at the Department of Psychiatry, First Affiliated Hospital, Zhejiang University School of Medicine, with a recruitment period from May 2022 through March 2023. This trial used a rigorous RCT design to investigate the potential therapeutic effects of combining tDCS and rTMS on cognitive function in patients diagnosed with BD.

**FIGURE 1:**
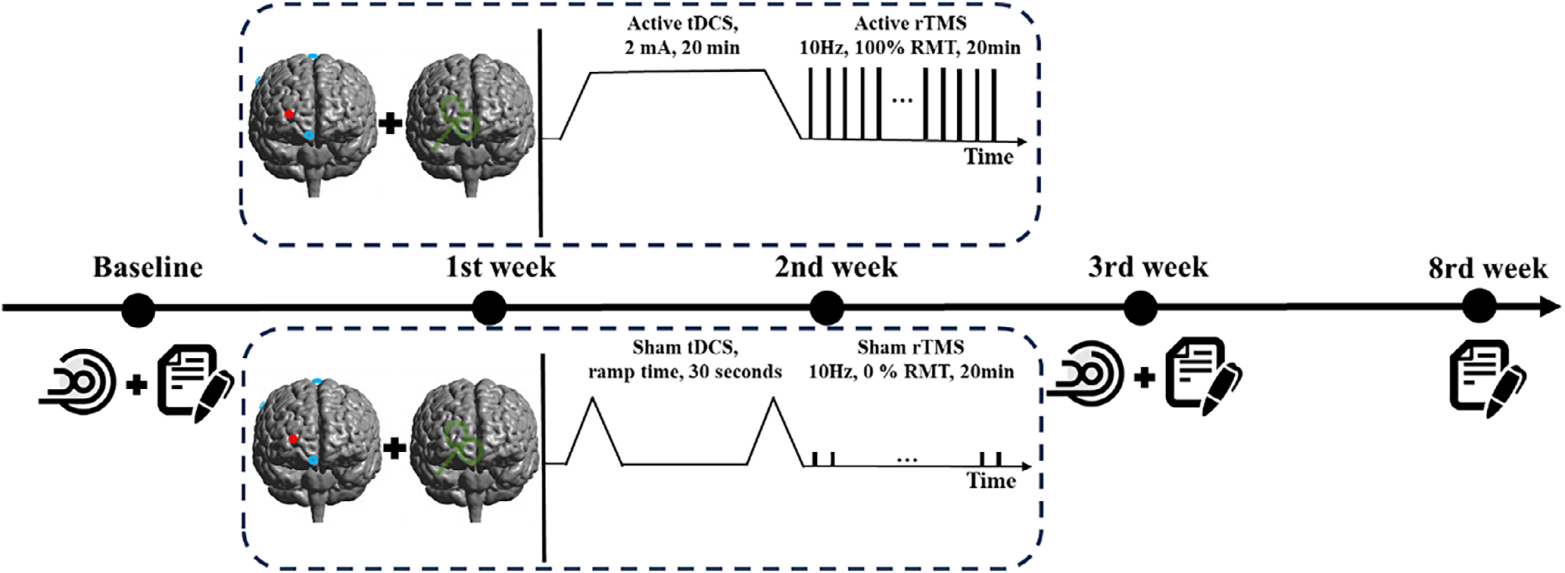
Electromagnetic stimulation paradigm. All patients underwent a 3‐week intervention of combined electromagnetic stimulation. Scale assessments and MRI data collection were performed before and after the intervention, followed by scale assessments at the eighth week.

Patients were randomly assigned to one of three groups: Group A (active tDCS combined with active rTMS), Group B (sham tDCS combined with active rTMS), and Group C (active tDCS combined with sham rTMS) (see Figure 1) through a random process using computer‐generated random numbers. Importantly, the patients and researchers were both blinded to the allocation results throughout the study. The patients were explicitly instructed not to engage in discussions regarding their treatment with fellow clinic attendees. All the participants received a 3‐week treatment regimen with five stimulation sessions per week, involving a total of 15 sequential tDCS combined neuronavigated rTMS during the whole study. The V1 areas functionally connected with the DLPFC was set as the stimulation target. Our initial procedure involved tDCS, which was subsequently followed by navigated rTMS. Throughout the trial, all participants consistently adhered to their primary medication regimen.

Ethical approval for this study was obtained from the Clinical Research Ethics Committee of the First Affiliated Hospital, Zhejiang University School of Medicine (Approval number: IIT20210036C‐R1). Before participation, all participants provided informed consent, ensuring their understanding of the objectives, procedures, and potential risks of this study. Participant confidentiality and well‐being was upheld throughout the study.

### 2.2 Participants

Participants in this study were required to meet the inclusion criteria as follows: (i) age range of 16 to 65 years; (ii) BD diagnosis established by two proficient psychiatrists, according to the Diagnostic and Statistical Manual of Mental Disorders, Fifth Edition (*DSM‐5*) and matched Mini‐International Neuropsychiatric Interview (M.I.N.I.); (iii) sustained and unchanged medication for at least 3 months; (iv) self‐reported cognitive decline assessed by a Perceived Deficits Questionnaire‐Depression (PDQ‐D) score of ≥ 17; (v) right‐handedness; and (vi) educational attainment of ≥ 9 years.

Exclusion criteria were established as follows: (i) concurrent presence of any other mental disorder defined in *DSM‐5*; (ii) historical documentation of significant neurological conditions, including epilepsy or traumatic brain injury; (iii) presence of substantial and unstable medical conditions, spanning diabetes or cardiovascular, hematological, endocrine, liver, or kidney disorders; (iv) prior history of substance or alcohol abuse; (v) prior history of suicide attempts or self‐reported suicidal ideation; (vi) pregnant or breastfeeding status; (vii) individuals experiencing achromatopsia, hypochromatopsia, or dysaudia; and (viii) contraindications hindering participation in the magnetic resonance imaging (MRI) scanning.

### 2.3 MRI Scanning

The MRI data were collected using a 3.0 Tesla scanner (GE, SIGNA) at the First Affiliated Hospital, Zhejiang University School of Medicine. High‐resolution T1‐weighted images were acquired using MPRAGE sequence with the following parameter settings: repetition time (TR) of 7.1 ms, echo time (TE) of 2.9 ms, flip angle of 8°, a field of view (FOV) of 260 × 260 mm^2^, a matrix size of 256 × 256, and a slice thickness of 1.0 mm. These individual anatomical data serve the purpose of localizing navigated rTMS. Functional images were obtained using an echo planar imaging sequence with the following parameters: TR = 1800 ms, TE = 30 ms, FOV = 240 × 240 mm^2^, inter‐slice gap = 0.8 mm, matrix size = 64 × 64, number of slices = 28, slice thickness = 4 mm, and 180 time points. During scanning, participants were instructed to keep their eyes closed. To minimize head motion, foam cushions were strategically placed on either side of the head, and earplugs were utilized to mitigate noise.

### 2.4 fMRI Data Preprocess and Graph Theory Analysis

The fMRI data preprocessing was performed using Data Processing Assistant for Resting‐State fMRI (DPARSF) Advanced Edition V5.4 in MATLAB 2020. The key preprocessing steps included segmenting MRI data into 10 periods, discarding the first 10 noisy time points, and synchronizing slice timing. Head movement exceeding 3 mm or 3° resulted in exclusion. Functional images were normalized to MNI space using T1‐based segmentation and registration. Spatial smoothing was conducted with an 8 mm × 8 mm × 8 mm Gaussian kernel. Linear drift was removed using linear regression to mitigate the effects of thermal noise. Band‐pass filtering (0.01–0.1 Hz) was applied to eliminate physiological noise.

Using DPARSF software, the brain was divided into regions based on the AAL116 template, treating each region as a network node. Pearson correlation coefficients of the time series were computed to construct brain network matrices, which were then Fisher *Z*‐transformed for subsequent statistical analysis. Graph theory analysis was performed using GRETNA software, calculating global and nodal metrics of the brain network matrices for both the base and post groups over a sparsity range of 0.01 < *S* < 0.35 with a step size of 0.01. Global metrics included small‐world index, normalized characteristic path length, characteristic path length, clustering coefficient, global efficiency, local efficiency, and network efficiency. Nodal metrics included nodal efficiency, nodal local efficiency, nodal clustering (NC) coefficient, nodal shortest path, betweenness centrality, and degree centrality (DC).

### 2.5 Treatment Protocol

As shown in Figure 1, each participant was enrolled in a comprehensive 3‐week treatment regimen, which encompassed a total of 15 successive sessions involving both rTMS and tDCS. The administration of rTMS was initiated 30 min after the completion of each tDCS session. It is noteworthy that the rTMS and tDCS interventions were directed to target the left V1 brain regions, functionally connected with the left DLPFC, consistent with our prior study [27].

The NIBS treatment was conducted by a skilled technician. The transcranial electrical stimulation device (Starstim, Neuroelectrics, Barcelona, Spain) was utilized to deliver a 20‐min stimulation current to participants during each session. Circular Ag/AgCl electrodes (PISTIM, Neuroelectrics, Barcelona, Spain) were utilized as the stimulating electrodes, with an electrode size of 3.14 cm^2^. Conductive gel (HD‐GEL, Soterix, New Jersey, USA) was applied as the conducting medium. For active tDCS protocol, the stimulation montage (PO3, 2 mA; FT7, −0.6 mA; CZ, −0.5 mA; Iz, −0.9 mA) was applied for 20 min including a 30 s ramp up to 2 mA at the start and a 30 s ramp down to 0 mA at the end. During the sham stimulation, the current was initially gradually increased over the first 30 s to replicate the initial sensation commonly experienced with tDCS. Subsequently, a 30‐s ramp‐down was implemented.

The rTMS procedures utilized the Magstim Rapid2 rTMS device (The Magstim Company, Whitland, UK) using a figure‐of‐eight coil configuration. For Stimulating target selection of neuronavigated rTMS [27], one region of interest (ROI) was defined in the DLPFC according to previously reported coordinates from earlier studies on BD (−32, 42, 32) [33, 34]. The DLPFC seeds were identified by 9‐mm spheres centered on the coordinates (−32, 42, 32) and were used to construct the FC maps. The above coordinate was used to identify the optimized TMS targeting coordinates in the V1 region using the computed seed‐based FC with the a priori identified ROIs in the DLPFC of 30 healthy subjects. The V1 stimulation sites were first determined according to their voxel‐wise FC with the previously defined ROIs in the DLPFC. The DLPFC was significantly anti‐correlated with V1 (6, −63, 15) with *r* = −0.179. These coordinates in V1 were then chosen as the rTMS targets. We used the Black Dolphin Navigation Robot (S‐50, a sub‐millimeter smart positioning system, Solide Medical Sci. & Tech. Co. Ltd., Xi'an, Shaanxi, China) with a figure‐of‐8 coil (Yingchi Tech, Shenzhen, China) to perform the rTMS. An infrared camera and a 3D‐printed three‐dimensional individual mask were used for precise navigation of the coil over the target area under real‐time visualization.

Each daily rTMS session encompassed a regimen of 60 five‐second trains, operating at a frequency of 10 Hz, administered at an intensity set to 110% of the participant's resting motor threshold. These trains were organized with inter‐train intervals of 20 s, thus accumulating to a total of 3000 pulses per treatment session. For the sham intervention, the positioning of the coil was meticulously replicated, and precisely targeted at the same anatomical locus. However, the intervention involved a simulated experience of scalp sensations akin to active rTMS, accompanied by the replication of auditory cues akin to the actual procedure. Importantly, this sham procedure did not involve the actual application of the magnetic field.

### 2.6 Cognitive Function and Emotional Assessment

Two trained and impartial psychiatrists conducted all psychometric assessments. Participants underwent evaluations at three‐time points: baseline (week 0, w0), end‐treatment (week 3, w3), and follow‐up (week 8, w8). THINC‐integrated tool (THINC‐it) was used to assess cognitive function. This set of assessment tools has been validated internationally and is an effective and sensitive tool for detecting cognitive impairment in affective disorders, assessing cognitive problems such as episodic memory, attention skills, and executive function in patients with BD [35, 36]. The objective test included Spotter, Symbol Check, Code breaker, Trails, and the subjective part of the test was a 5‐item questionnaire for cognitive impartment (PDQ‐5‐D). In our study, the THINC‐it test was conducted in the order of PDQ‐5‐D, Spotter, Symbol Check, Codebreaker, and Trails. Additional evaluations comprised the alteration in the 17‐item Hamilton Depression Rating Scale (HDRS‐17) and the Young Mania Rating Scale (YMRS). Any adverse events encountered were meticulously documented.

### 2.7 Statistical Analysis

The collected scores from the assessment tools were subjected to statistical analysis using repeated measures analysis of covariance (ANOVA) and paired *t*‐tests. Repeated measures ANOVA was used to assess within‐group and between‐group differences across the three‐time points (w0, w3, and w8). Paired *t*‐tests were used to investigate specific changes between baseline and post‐treatment or follow‐up assessments.

This study used MATLAB 2020b, SPM12, and DPABI for statistical analysis. SPM12 was used to conduct paired *t*‐tests to examine variations in fALFF, regional homogeneity (ReHo), and FC between groups. The DPABI software [37], in conjunction with Gaussian Random Field theory, was utilized to correct for multiple comparisons in the paired *t*‐test results. This correction included a voxel threshold of *p* < 0.01 and a clump threshold of *p* < 0.05.

## 3 Results

### 3.1 Patient Demographics

**FIGURE 2:**
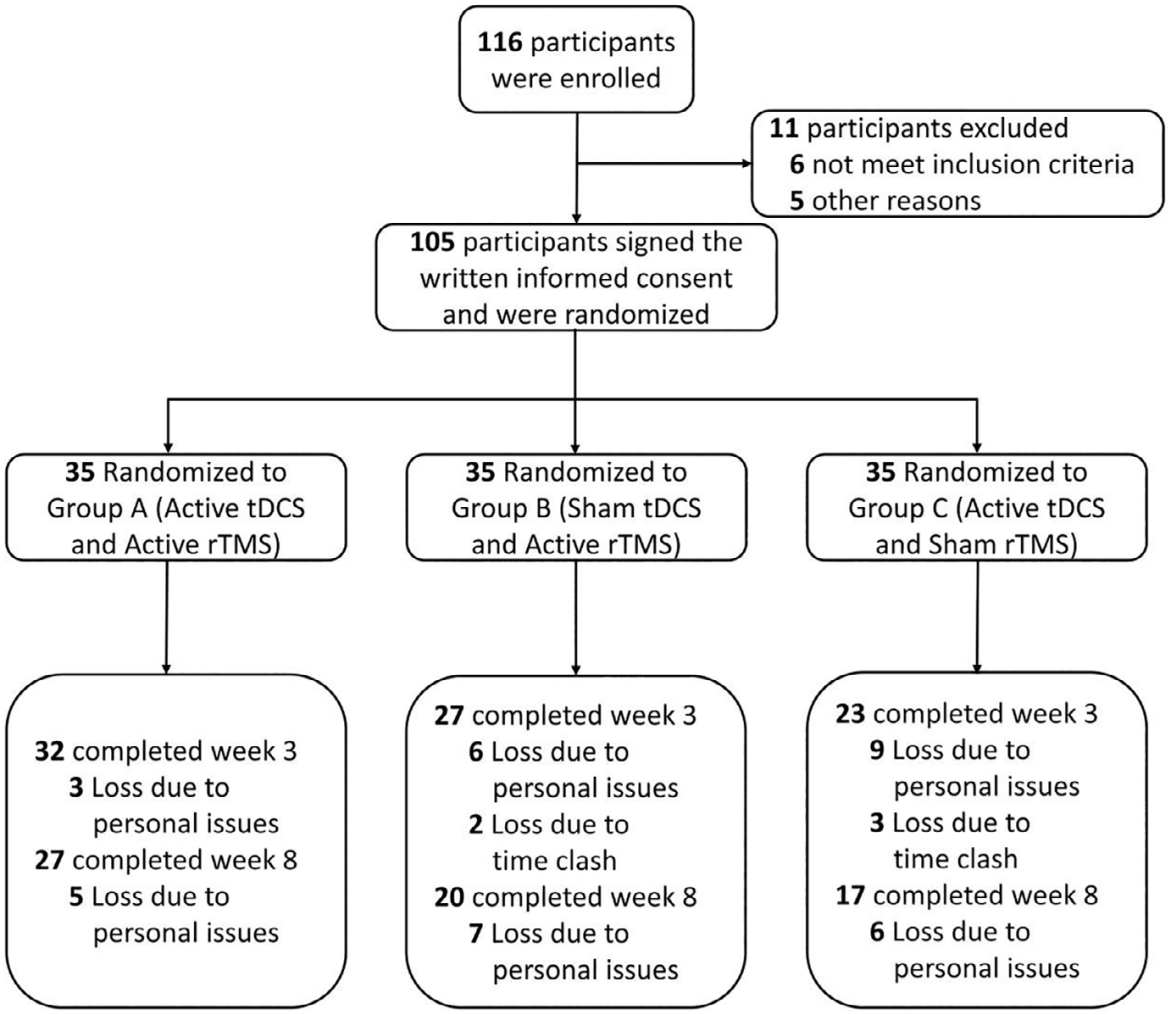
Flow diagram of participant selection and intervention.

As shown in Figure 2, the study recruited 116 participants, of which 11 were excluded. Eventually, a total of 105 BD patients were enrolled, and randomly assigned into across three treatment groups: Group A (active tDCS‐active rTMS (*n* = 35)), Group B (sham tDCS‐active rTMS (*n* = 35)), and Group C (active tDCS‐sham rTMS (*n* = 35)). Of these 105 participants, 82 received all 15 planned sessions of tDCS‐rTMS and completed the week‐0 and week‐3 assessments (32 participants in Group A, 27 in Group B, and 23 in Group C). The primary reasons for participants not completing the full trial protocol were related to their individual hospitalization durations, time conflicts, and improvements in their clinical condition. The demographic characteristics of these participants, including age, gender, and baseline symptom severity, were comparable across the groups (see Table 1).

**TABLE 1:** Demographic and clinical characteristics of the participants at baseline.

Characteristic	Group A (active tDCS‐active rTMS)	Group B (sham tDCS‐active rTMS)	Group C (active tDCS‐sham rTMS)	Analysis *F*/*χ* ^2^	*p*
Age	23.38 ± 8.06	25.74 ± 8.66	20.43 ± 6.06	2.896	0.061
Sex (male/female)	10/22	10/17	6/17	0.693	0.707*
Education years	13.69 ± 3.09	14.33 ± 2.70	12.43 ± 2.19	3.052	0.053
Disease course (months)	46.38 ± 36.04	67.89 ± 72.30	52.00 ± 45.59	1.259	0.290
Body mass index	23.48 ± 4.72	23.10 ± 4.11	23.67 ± 5.12	1.020	0.903
YMRS score	2.97 ± 2.76	4.26 ± 3.56	5.52 ± 5.32	2.925	0.059
HDRS‐17 score	9.38 ± 6.69	8.33 ± 4.67	10.52 ± 6.49	0.815	0.446
PDQ‐5‐D	9.34 ± 5.02	9.30 ± 5.61	11.09 ± 4.73	0.975	0.382

*Note: p* value: * indicates the chi‐square test result.

### 3.2 Cognitive Function

**FIGURE 3:**
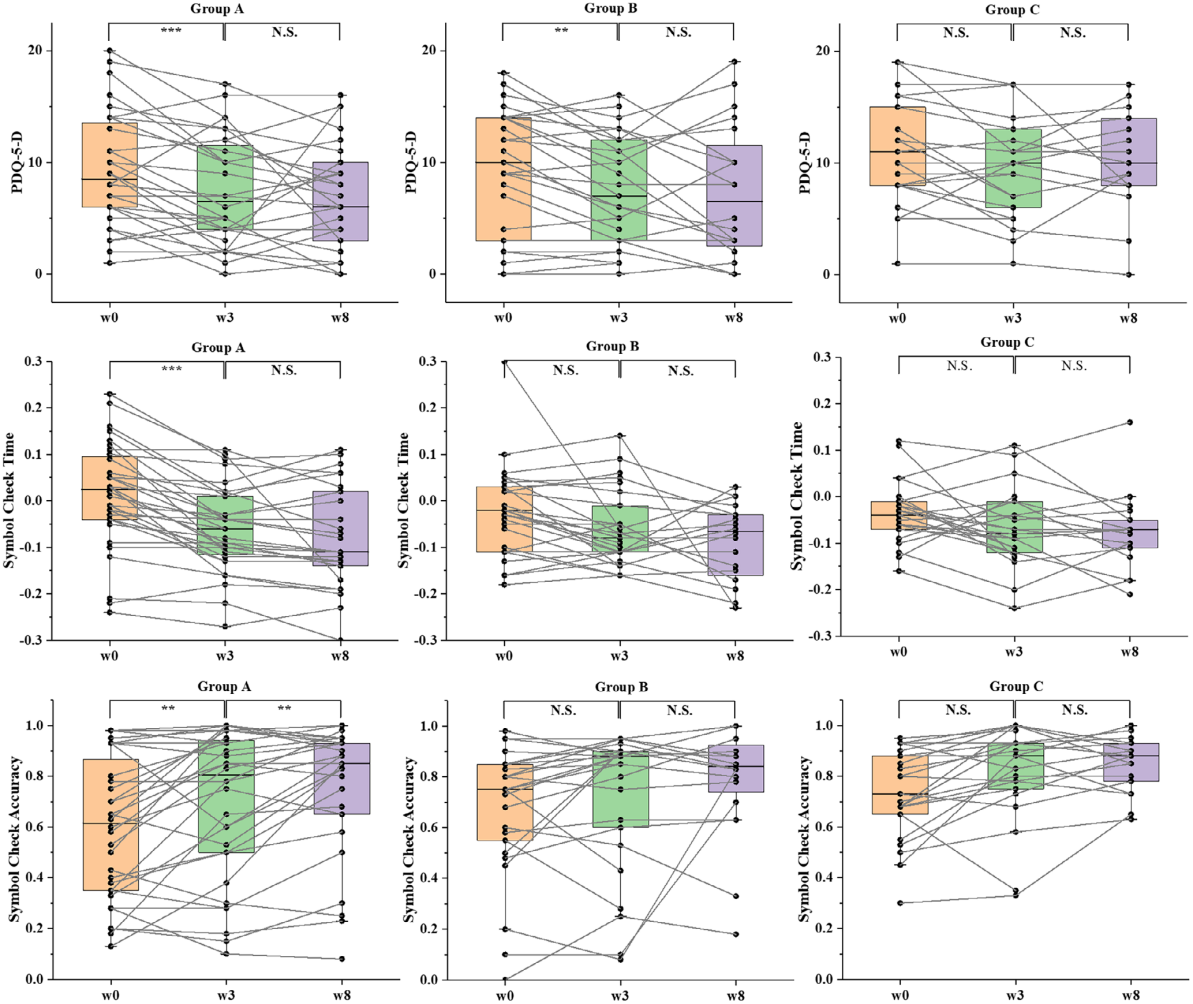
Pairwise comparisons of the PDQ‐5‐D, Symbol Check Time, and Symbol Check Accuracy scores for the three groups (Group A (active tDCS‐active rTMS), Group B (sham tDCS‐active rTMS), and Group C (active tDCS‐sham rTMS)). Each data point represents participants with recorded scores at that time point. N.S., non‐significant; w0, Week 0; w3, Week 3; w8, Week 8; **p < 0.01, ***p < 0.001.

No significant interactions between time and intervention type (Groups A, B, and C) were observed using a repeated‐measures ANOVA approach. The application of active rTMS in conjunction with active tDCS resulted in markedly enhanced cognitive function when compared to the other treatment combinations. Statistical analysis showed that Group A exhibited significantly greater improvements in cognitive function (Symbol Check Time and Symbol Check Accuracy) compared to both Group B and Group C at week 3. Group A also showed sustained significance in Symbol Check Accuracy at week 8 (*p* < 0.01) (Table 2, Figure 3). In addition, a significant improvement in cognitive impairment, as assessed by the PDQ‐5‐D, was observed in both Group A and Group B between week 0 and week 3. These findings suggested a synergistic effect of combining rTMS and tDCS on cognitive enhancement in BD patients.

**TABLE 2:** Outcomes on six items of THINC‐it cognitive function scores for participants.

Characteristic	Group A (active tDCS‐active rTMS)			*p*1	*p*2	Group B (sham tDCS‐active rTMS)			*p*1	*p*2	Group C (active tDCS‐sham rTMS)			*p*1	*p*2
	w0	w3	w8			w0	w3	w8			w0	w3	w8		
PDQ‐5‐D	9.34 ± 5.02	7.38 ± 4.74	7.00 ± 4.65	< 0.001	> 0.01	9.30 ± 5.61	7.48 ± 4.81	7.25 ± 6.03	< 0.01	> 0.01	11.09 ± 4.73	9.65 ± 4.46	10.18 ± 4.56	> 0.01	> 0.01
Spotter CRT	−0.28 ± 0.19	−0.30 ± 0.08	−0.33 ± 0.09	> 0.01	> 0.01	−0.30 ± 0.08	−0.27 ± 0.19	−0.30 ± 0.09	> 0.01	> 0.01	−0.30 ± 0.12	−0.30 ± 0.12	−0.32 ± 0.12	> 0.01	> 0.01
Symbol check (time)	0.01 ± 0.11	−0.06 ± 0.09	−0.09 ± 0.12	< 0.001	> 0.01	−0.02 ± 0.10	−0.06 ± 0.08	−0.09 ± 0.08	> 0.01	> 0.01	−0.03 ± 0.07	−0.07 ± 0.08	−0.07 ± 0.08	> 0.01	> 0.01
Symbol check (accuracy)	0.59 ± 0.28	0.70 ± 0.29	0.75 ± 0.26	< 0.01	< 0.01	0.68 ± 0.25	0.73 ± 0.27	0.79 ± 0.22	> 0.01	> 0.01	0.72 ± 0.18	0.81 ± 0.19	0.84 ± 0.11	> 0.01	> 0.01
Codebreaker	58.50 ± 18.07	61.44 ± 15.68	64.59 ± 19.45	> 0.01	> 0.01	54.78 ± 16.71	59.96 ± 14.00	63.60 ± 15.75	> 0.01	> 0.01	57.65 ± 11.25	64.00 ± 16.23	63.65 ± 11.69	> 0.01	> 0.01
Trails	23.41 ± 10.43	24.76 ± 17.45	19.17 ± 5.67	> 0.01	> 0.01	24.11 ± 7.08	21.04 ± 7.31	19.40 ± 8.89	> 0.01	> 0.01	25.64 ± 8.98	22.50 ± 9.72	20.61 ± 6.17	> 0.01	> 0.01

*Note:* The *p*1 value indicates the paired *t*‐test result between the time of measurement (w0/w3), *p*2 value indicates the paired *t*‐test result between the time of measurement (w3/w8).

### 3.3 Depressive Symptoms

**FIGURE 4:**
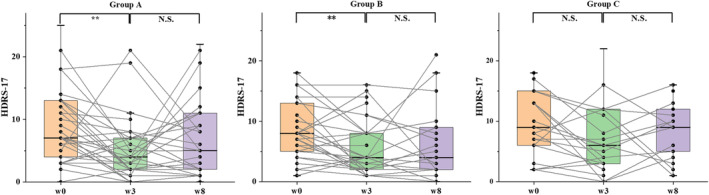
Pairwise comparisons of the depressive severity for the three groups. Group A (active tDCS‐active rTMS), Group B (sham tDCS‐active rTMS), and Group C (active tDCS‐sham rTMS). Each data point represents participants with recorded scores at that time point. N.S., non‐significant; w0, Week 0; w3, Week 3; w8, Week 8; **p < 0.01.

No statistically significant reduction in depressive symptoms measured with the HDRS‐17 was observed in Group C. However, both Group A and Group B demonstrated a significant improvement in the severity of depressive symptoms from baseline to week 3 (*p* < 0.01) (Figure 4). These results suggested that active tDCS and rTMS contribute significantly to the amelioration of residual depressive symptoms.

### 3.4 Adverse Effects

Adverse events were primarily evident in proximity to and following active rTMS and tDCS interventions. Among the cohort of 1 participant encountering adverse events, the prevailing nature was mild and transitory. Manifestations primarily encompassed mild cephalgia, slight dizziness, intermittent nausea, momentary somnolence, fleeting earache, and localized discomfort at the stimulation site.

### 3.5 Results of Graph Theory Analysis

**FIGURE 5:**
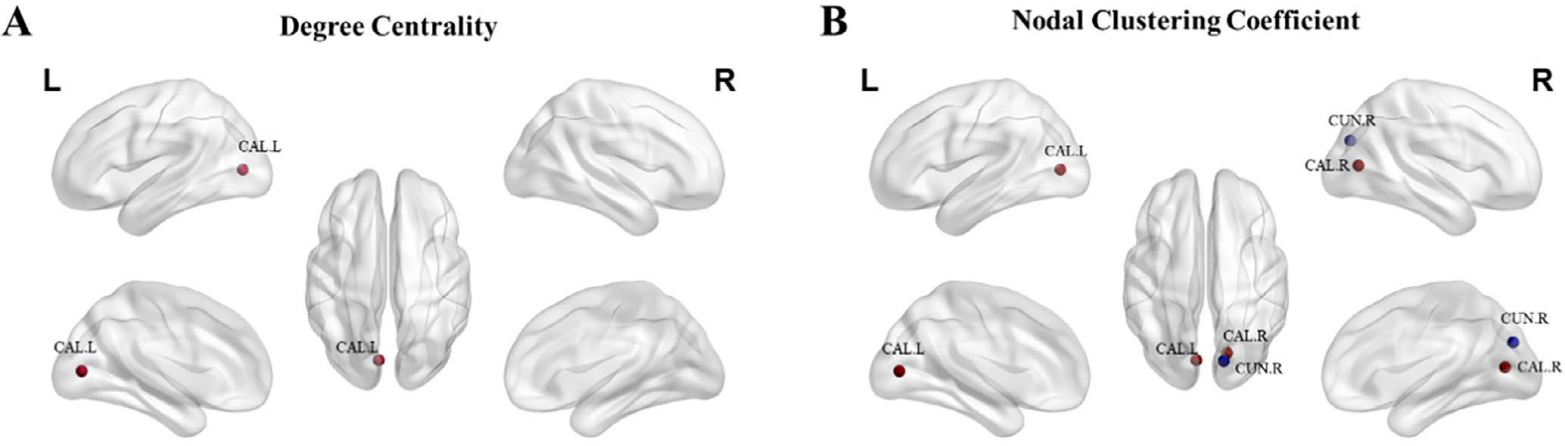
Changes in degree centrality (DC) and nodal clustering coefficient (NC) of VIS network‐related nodes after intervention. (A) The results of DC; (B) the results of NC. Red spheres indicate enhancement, while blue spheres indicate reduction. CAL‐L, left calcarine cortex; CAL‐R, right calcarine cortex; CUR‐R, right cuneus.

For the default mode network, DAN, and six other functional networks, there were no significant changes in global metrics across the three intervention groups. However, the node metrics of visual network (VIS) in Group A showed significant differences before and after the intervention (*p* < 0.05). The specific results are shown in Figure 5. Following combined electromagnetic stimulation, the VIS brain region exhibited varying degrees of increase in DC and NC coefficient in the cortex surrounding the left calcarine sulcus, with a downward trend in NC observed in the right cuneus. DC represents the number of edges or nodes directly connected to a given node, whereas NC reflects the degree of connectivity between a node and its neighboring nodes, with higher values indicating greater modularization of the node.

## 4 Discussion

To our knowledge, it was the first study to combine the navigated rTMS and tDCS to modulate the cognitive function of BD patients. Our findings signify the potential benefits of this novel intervention, with a notable emphasis on safety considerations, for enhancing cognitive function in BD patients.

Our results unveil a marked enhancement in cognitive function with the simultaneous application of active rTMS and tDCS in BD patients. This observed cognitive improvement is consistent with prior electrophysiological studies, which suggest that the combination of these two NIBS techniques produces synergistic cortical effects [14, 38, 39]. Recent studies have also provided preliminary clinical evidence supporting the advantages of combined rTMS and tDCS in alleviating depressive symptoms [40, 41], further substantiating the efficacy of this multimodal intervention approach. Conventional single‐mode TMS targeting V1 has been shown to enhance cognitive abilities, but its long‐term maintenance effect is limited [27]. The combined stimulation approach proposed in this article extends the post‐stimulation effect to some extent, particularly for specific cognitive indicators like symbol check accuracy. The synergy could arise from the complementary mechanisms of rTMS and tDCS, where rTMS induces neuronal firing through magnetic pulses while tDCS modulates resting membrane potentials, potentially enhancing synaptic plasticity and cognitive processes. The substantial cognitive improvements observed in our study parallel and extend existing evidence, bolstering the notion that combined rTMS and tDCS interventions hold significant promise in enhancing cognitive function.

In addition, numerous studies have investigated the enhancement of cognitive function through tDCS, and most relevant interventions have been concentrated on the prefrontal and parietal brain regions [42, 43]. There is substantial evidence supporting the notion that tDCS applied to the prefrontal cortex can enhance performance in tasks involving task switching, dual tasking, updating, and inhibition [44, 45, 46]. tDCS also demonstrated the capability to improve working memory performance in the healthy older adult population [47]. Although Group C in this study did not have statistical significance for THINC‐it cognitive function scores, it demonstrated the potential for improving cognition with BD by targeting the V1 cortex of the occipital lobe through tDCS.

A pivotal aspect of our study is the meticulous consideration of safety throughout the intervention. The successful completion of the treatment by a substantial proportion of participants across all three groups attests to the feasibility and safety of the combined rTMS‐active tDCS intervention. This aligns with our focus on patient well‐being, corroborating with prior studies that have similarly prioritized safety in the application of NIBS techniques. Our findings contribute to the growing body of evidence highlighting the safety of these interventions, further endorsing their potential as adjunctive therapeutic strategies.

The results of this study demonstrate that combined electromagnetic stimulation leads to increased brain network activity in the VIS of BD patients. Specifically, there is an increase in the DC and NC coefficient of the calcarine sulcus, indicating enhanced information transmission efficiency and stronger connectivity with surrounding nodes. This suggests that the combined stimulation improves the function of visual information processing and enhances cooperation with other visual cortical areas in BD patients.

The calcarine sulcus, as an essential part of the primary visual cortex, plays a crucial role in visual processing [48]. Previous research has shown that BD patients exhibit lower multiscale entropy (MSE) in the calcarine sulcus, precuneus, and cerebellum compared to control groups, while regions such as the middle cingulate cortex, thalamus, hippocampus, middle temporal gyrus, and middle frontal gyrus show increased MSE, indicating higher complexity in these areas [49]. This previous study also explored the correlation between MSE and ReHo and ALFF (amplitude of low‐frequency fluctuations), finding a significant positive correlation between MSE and these indices in the calcarine sulcus. This confirms that BD patients have lower intensity of spontaneous neural activity and lower ReHo in the calcarine sulcus compared to control groups, suggesting impaired or insufficient engagement of this region in spontaneous neural activity, leading to diminished visual and cognitive processing capabilities. These findings imply that the dysfunction in neural networks related to emotional regulation and visual information integration may be linked to the reduced spontaneous neural activity in the calcarine sulcus observed in BD patients [50]. Therefore, it can be speculated that combined electromagnetic stimulation might induce neuroplastic changes in the visual cortex, altering visual information processing, and enhancing neuronal connectivity. This, in turn, could increase the activity of the cortex surrounding the calcarine sulcus, helping patients integrate visual and emotional information more effectively.

The implications of our study are multifaceted. The potent cognitive enhancement observed with the combined rTMS and tDCS intervention highlights the potential for advanced cognitive rehabilitation strategies in patients with BD. In considering future directions, expanding the sample size and extending the follow‐up period could yield a more comprehensive understanding of the sustained effects of these interventions. Furthermore, for the intervention protocols, individualized transcranial electromagnetic simulation models can be constructed using subject‐specific magnetic resonance structural information. This could allow for optimization of the coil placement and angle for transcranial magnetic stimulation, as well as the optimization of transcranial electrical stimulation parameters. Finally, exploring the interplay between individual variability in response and neural mechanisms underlying combined NIBS effects could yield further insights into tailored interventions.

Although this study provides valuable insights into the potential benefits of combining active rTMS with active tDCS for enhancing neuropsychological outcomes in individuals with BD, several limitations warrant consideration. First, the sample size was moderate, potentially limiting the generalizability of our findings to a broader BD population. Additionally, our clinical trial did not include a dual‐sham control group for electromagnetic interventions, making it challenging to evaluate the placebo effect. Second, the relatively short follow‐up period of 8 weeks leaves room for future investigations to explore the durability and long‐term effects of the observed enhancements. Additionally, variations in stimulation protocols (such as rTMS followed by tDCS), target regions, and participant characteristics across different studies could influence the interpretation of our results. Lastly, the study's focus on cognitive function and depressive symptoms might benefit from the inclusion of other relevant outcome measures to provide a more comprehensive evaluation of the intervention's impact. Addressing these limitations in future research will offer a more nuanced understanding of the efficacy and applicability of combined rTMS and tDCS interventions in the context of BD.

## 5 Conclusion

The present study highlights the potential benefits of applying combined rTMS and tDCS to the V1‐DLPFC FC in improving cognitive function and depressive symptoms among BD patients. The findings suggest that this novel intervention holds promise for enhancing neuropsychological outcomes in this population, with a notable focus on safety considerations. Moreover, the observed increased activity in the calcarine sulcus following stimulation indicates potential neuroplastic changes in the visual cortex that may underlie the cognitive improvements seen in BD patients.

## Author Contributions

S. Hu, S. Zhang, and F. Wang had full access to all of the data in the study and take responsibility for the integrity of the data and the accuracy of the data analysis. Concept and design: S. Hu, S. Zhang, and F. Wang. Acquisition, analysis, or interpretation of data: All authors. Drafting of the manuscript: H. Zhou, M. Wang. Critical review of the manuscript for important intellectual content: All authors. Statistical analysis: H. Zhou, M. Wang. Obtained funding: S. Hu. Administrative, technical, or material support: H. Zhou, M. Wang, T. Xu, X. Zhang, X. Zhao, D. Wang, and J. Lai. Supervision: S. Hu, S. Zhang, and F. Wang.

## Conflicts of Interest

The authors declare no conflicts of interest.

## Supporting information


Data S1.

## Data Availability

The full trial protocol can be available in the Supporting Information.
