# Supplementary material for: Cognitive Remediation in Patients With Bipolar Disorder: A Randomized Trial by Sequential tDCS and Navigated rTMS Targeting the Primary Visual Cortex
Source: CNS Neurosci Ther. 2024 Dec 20;30(12):e70179. doi: 10.1111/cns.70179 (PMC11659637; doi:10.1111/cns.70179)
Supplement: Supplementary file 1 — Data S1. [file CNS-30-e70179-s001.docx]

**Table S1.** Demographic and Clinical Characteristics of the participants at baseline

| **Characteristic** | **Group A**  **(Active tDCS-Active rTMS)** | **Group B**  **(Sham tDCS-Active rTMS)** | **Group C**  **(Active tDCS-Sham rTMS)** | **Analysis F/χ2** | ***P*** | ***f/Cramér's V*** |
| --- | --- | --- | --- | --- | --- | --- |
| **Age** | 23.38±8.06 | 25.74±8.66 | 20.43±6.06 | 2.896 | 0.061 | 0.27 |
| **Sex (Male/Female)** | 10/22 | 10/17 | 6/17 | 0.693 | 0.707* | 0.09* |
| **Education years** | 13.69±3.09 | 14.33±2.70 | 12.43±2.19 | 3.052 | 0.053 | 0.28 |
| **Disease course (Months)** | 46.38±36.04 | 67.89±72.30 | 52.00±45.59 | 1.259 | 0.290 | 0.18 |
| **Body mass index** | 23.48±4.72 | 23.10±4.11 | 23.67±5.12 | 1.020 | 0.903 | 0.05 |
| **YMRS score** | 2.97±2.76 | 4.26±3.56 | 5.52±5.32 | 2.925 | 0.059 | 0.27 |
| **HDRS-17 score** | 9.38±6.69 | 8.33±4.67 | 10.52±6.49 | 0.815 | 0.446 | 0.14 |
| **PDQ-5-D** | 9.34±5.02 | 9.30±5.61 | 11.09±4.73 | 0.975 | 0.382 | 0.16 |

* indicates the chi-square test result.

**Table S2**. Outcomes on six items of THINC-it cognitive function scores for participants of group A

| **Characteristic** | **Group A**  **(Active tDCS-Active rTMS)** | | | ***P1*** | ***Cohen’s d*** | ***P2*** | ***Cohen’s d*** |
| --- | --- | --- | --- | --- | --- | --- | --- |
|  | **w0** | **w3** | **w8** |  |  |  |  |
| **PDQ-5-D** | 9.34±5.02 | 7.38±4.74 | 7.00±4.65 | <0.001 | 0.40 | >0.01 | 0.08 |
| **Spotter CRT** | -0.28±0.19 | -0.30±0.08 | -0.33±0.09 | >0.01 | 0.14 | >0.01 | 0.35 |
| **Symbol Check (Time)** | 0.01±0.11 | -0.06±0.09 | -0.09±0.12 | <0.001 | 0.70 | >0.01 | 0.28 |
| **Symbol Check (Accuracy)** | 0.59±0.28 | 0.70±0.29 | 0.75±0.26 | <0.01 | 0.39 | <0.01 | 0.18 |
| **Codebreaker** | 58.50±18.07 | 61.44±15.68 | 64.59±19.45 | >0.01 | 0.17 | >0.01 | 0.18 |
| **Trails** | 23.41±10.43 | 24.76±17.45 | 19.17±5.67 | >0.01 | 0.09 | >0.01 | 0.43 |

The *P1* value indicates the paired t-test result between the time of measurement (w0/w3), *P2* value indicates the paired t-test result between the time of measurement (w3/w8).

**Table S3**. Outcomes on six items of THINC-it cognitive function scores for participants of group B

| **Characteristic** | **Group B**  **(Sham tDCS-Active rTMS)** | | | ***P1*** | ***Cohen’s d*** | ***P2*** | ***Cohen’s d*** |
| --- | --- | --- | --- | --- | --- | --- | --- |
|  | **w0** | **w3** | **w8** |  |  |  |  |
| **PDQ-5-D** | 9.30±5.61 | 7.48±4.81 | 7.25±6.03 | <0.01 | 0.35 | >0.01 | 0.04 |
| **Spotter CRT** | -0.30±0.08 | -0.27±0.19 | -0.30±0.09 | >0.01 | 0.21 | >0.01 | 0.20 |
| **Symbol Check (Time)** | -0.02±0.10 | -0.06±0.08 | -0.09±0.08 | >0.01 | 0.44 | >0.01 | 0.38 |
| **Symbol Check (Accuracy)** | 0.68±0.25 | 0.73±0.27 | 0.79±0.22 | >0.01 | 0.19 | >0.01 | 0.24 |
| **Codebreaker** | 54.78±16.71 | 59.96±14.00 | 63.60±15.75 | >0.01 | 0.34 | >0.01 | 0.22 |
| **Trails** | 24.11±7.08 | 21.04±7.31 | 19.40±8.89 | >0.01 | 0.43 | >0.01 | 0.20 |

The *P1* value indicates the paired t-test result between the time of measurement (w0/w3), *P2* value indicates the paired t-test result between the time of measurement (w3/w8).

**Table S4**. Outcomes on six items of THINC-it cognitive function scores for participants of group C

| **Characteristic** | **Group C**  **(Active tDCS-Sham rTMS)** | | | ***P1*** | ***Cohen’s d*** | ***P2*** | ***Cohen’s d*** |
| --- | --- | --- | --- | --- | --- | --- | --- |
|  | **w0** | **w3** | **w8** |  |  |  |  |
| **PDQ-5-D** | 11.09±4.73 | 9.65±4.46 | 10.18±4.56 | >0.01 | 0.31 | >0.01 | 0.12 |
| **Spotter CRT** | -0.30±0.12 | -0.30±0.12 | -0.32±0.12 | >0.01 | 0.00 | >0.01 | 0.17 |
| **Symbol Check (Time)** | -0.03±0.07 | -0.07±0.08 | -0.07±0.08 | >0.01 | 0.53 | >0.01 | 0.00 |
| **Symbol Check (Accuracy)** | 0.72±0.18 | 0.81±0.19 | 0.84±0.11 | >0.01 | 0.49 | >0.01 | 0.19 |
| **Codebreaker** | 57.65±11.25 | 64.00±16.23 | 63.65±11.69 | >0.01 | 0.45 | >0.01 | 0.02 |
| **Trails** | 25.64±8.98 | 22.50±9.72 | 20.61±6.17 | >0.01 | 0.34 | >0.01 | 0.23 |

The *P1* value indicates the paired t-test result between the time of measurement (w0/w3), *P2* value indicates the paired t-test result between the time of measurement (w3/w8)
